# The Association Between Civil Legal Needs After Incarceration, Psychosocial Stress, and Cardiovascular Disease Risk Factors

**DOI:** 10.1017/jme.2024.26

**Published:** 2023

**Authors:** Benjamin Lu, Kathryn Thomas, Solomon Feder, James Bhandary-Alexander, Jenerius Aminawung, Lisa B. Puglisi

**Affiliations:** 1. YALE LAW SCHOOL, NEW HAVEN, CT, USA; 2. SEICHE CENTER FOR HEALTH AND JUSTICE, YALE SCHOOL OF MEDICINE, NEW HAVEN, CT, USA

**Keywords:** Medical-Legal Partnerships, Cardiovascular Health

## Abstract

Many formerly incarcerated people have civil legal needs that can imperil their successful re-entry to society and, consequently, their health. We categorize these needs and assess their association with cardiovascular disease risk factors in a sample of recently released people. We find that having legal needs related to debt, public benefits, housing, or healthcare access is associated with psychosocial stress, but not uncontrolled high blood pressure or high cholesterol, in the first three months after release.

## Background

Some 1.9 million people are incarcerated in prisons and jails on any given day in the United States.^1^ Each year, about 650,000 of them return to their communities from prison,^2^ and more than 7 million return from jail.^3^ Although they have exited the carceral system, their preceding arrests, criminal charges, terms of incarceration, and convictions can impose ongoing economic, social, and legal difficulties even after release. These so-termed collateral consequences are ubiquitous: The National Inventory of Collateral Consequences of Conviction notes 44,000 legal consequences arising from conviction alone, including voter disenfranchisement, housing restrictions, food stamp bans, and barriers to employment.^4^

Researchers have posited that collateral consequences harm the health of formerly incarcerated people,^5^ their families,^6^ and their communities.^7^ At the individual level, for example, research has found that people who present as recently released from incarceration when seeking an initial primary care appointment are less likely to be offered one than people who do not.^8^ And at the population level, geographic areas with higher rates of incarceration tend to have higher levels of morbidity and mortality.^9^ There is also evidence that the prevalence and severity of collateral consequences can vary across subpopulations. One field experiment, for example, found marked racial disparities in the impact of a criminal record on employment outcomes.^10^ Further research to identify which specific collateral consequences harm patient health, how they do so, and how best to mitigate their harms can inform medical and legal practitioners serving people recently released from incarceration.

This paper focuses on the potential associations between common civil legal needs after incarceration, psychosocial stress, and cardiovascular health. Previous studies have found that recent incarceration is associated with higher rates of stress and worse cardiovascular disease (CVD) outcomes, even after accounting for sociodemographic features and traditional CVD risk factors, like uncontrolled high blood pressure and uncontrolled high cholesterol, at baseline.^11^ Specifically, we investigate the role that civil legal needs arising from collateral consequences might play in explaining this association by analyzing data from a cohort of recently released people in Connecticut.

This paper focuses on the potential associations between common civil legal needs after incarceration, psychosocial stress, and cardiovascular health. Previous studies have found that recent incarceration is associated with higher rates of stress and worse cardiovascular disease (CVD) outcomes, even after accounting for sociodemographic features and traditional CVD risk factors, like uncontrolled high blood pressure and uncontrolled high cholesterol, at baseline. Specifically, we investigate the role that civil legal needs arising from collateral consequences might play in explaining this association by analyzing data from a cohort of recently released people in Connecticut.

## Methods

### Sample

Our data were collected as part of the Justice-Involved Individuals Cardiovascular Disease Epidemiology (JUSTICE) study, an ongoing prospective cohort study of formerly incarcerated individuals residing in New Haven, Bridgeport, and Hartford, Connecticut with known CVD risk factors.^12^ The study, which some authors of this paper began in 2019, aims to recruit 500 participants within three months of their release from jail or prison and follow them for 12 months. At baseline, the study assesses each participant for control of CVD risk factors through point-of-care testing that includes a blood lipid panel and hemoglobin A1C, a test of diabetes; direct blood pressure measurements; and detailed questions about diet, exercise, and smoking. It also measures participants’ prior exposure to incarceration-related policies like restrictive housing or solitary confinement, their psychosocial stress level on the Perceived Stress Scale,^13^ and their self-efficacy. After these baseline measurements are taken, participants’ psychosocial factors and clinical risk factors are re-evaluated every six months for a year. With these longitudinal data, the study aims to estimate the effects of incarceration on CVD risk factors and the paths via which these effects are mediated. Although the JUSTICE study is still ongoing, we analyze some of its baseline data here to assess early associations between civil legal needs, psychosocial stress, and cardiovascular risk factors shortly after release from incarceration.

### Civil Legal Needs (Exposure Variable)

In the baseline survey, participants were asked if they were experiencing 18 different post-incarceration civil legal needs. These included issues with wage garnishment, driver and occupational licenses, government benefits, living conditions, child support, and health insurance. Table 1 contains the full list of needs. The list was developed iteratively over the past decade through some authors’ work running a clinic within the Transitions Clinic Network,^14^ a consortium of primary care clinics focused on the health needs of people returning from incarceration, and directing a medical-legal partnership within the clinic.

**Table 1:**
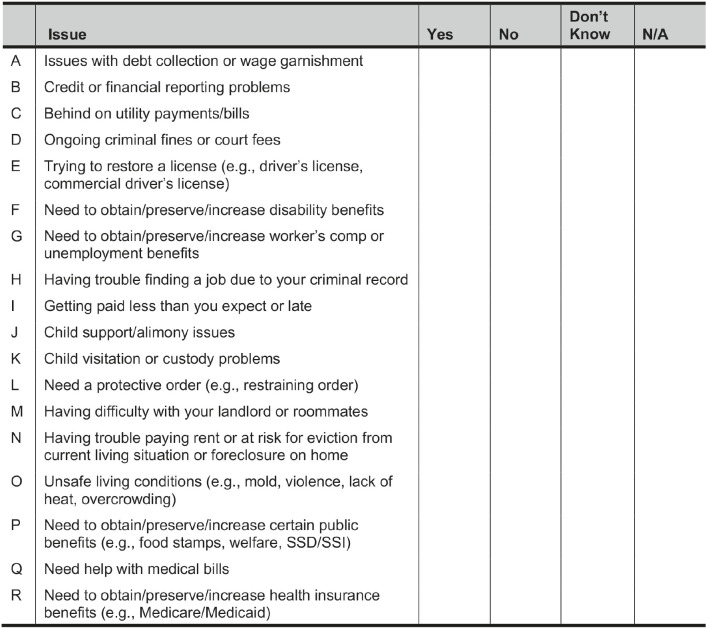
JUSTICE study civil legal needs screener.

### Factor Analysis

To use these data in subsequent analyses, we first conceptually organized the 18 civil legal needs into broader categories of related needs. We did so via an exploratory factor analysis of the data with varimax rotation, which seeks to identify the latent categories, or factors, that drive the observed patterns in participants’ civil legal needs. We decided a priori to keep factors with at least two civil legal needs. We checked the resulting factors against the broader literature on common civil legal needs and their plausible associations with health outcomes.^15^

### Outcome Variable

Our outcomes were binary indicators for the presence of moderate to severe psychosocial stress as measured by the Perceived Stress Scale,^16^ uncontrolled high blood pressure (greater than 140/90 mmHg), and uncontrolled total cholesterol (greater than 200 mg/dl). These outcomes were measured through point-of-care testing at baseline concurrently with the civil legal needs screener. Thus, the data as a whole are cross-sectional, measured at a single point in time within three months of participants’ release from incarceration.

### Analysis

We assessed the association between each latent factor and outcome. Specifically, for each latent factor, we compared the prevalence of each outcome at baseline between those who reported encountering at least one civil legal need in the latent factor and those who reported no civil legal needs in the latent factor. We used chi-squared tests to assess statistical significance to generate hypotheses for future analyses. All analyses were performed using SPSS version 26, and all statistical tests were two-tailed and used a p-value of 0.05 as a threshold for significance.

## Results

The dataset contained 345 participants. Most were male (*n* = 316; 91.6%), were non-Hispanic Black (*n* = 179; 51.9%), and lived in a transitional home (*n* = 267; 77.4%). See Table 2 for summary statistics of participants’ demographic characteristics, including age, gender, race and ethnicity, marital status, highest education achieved, insurance status, employment, average monthly income, and food insecurity. At baseline, 79 percent reported a prior diagnosis of hypertension, 60 percent reported a prior diagnosis of obesity, 47 percent reported a prior diagnosis of hyperlipidemia, and 29 percent reported a prior diagnosis of diabetes.

**Table 2:**
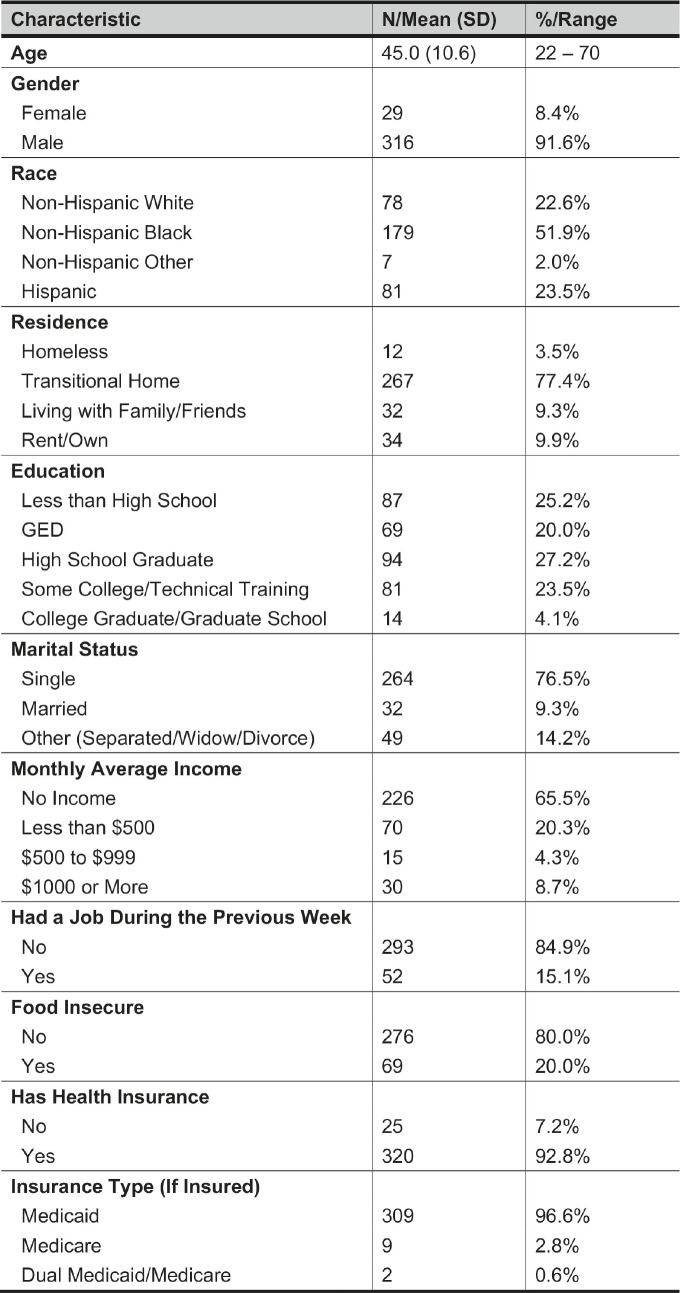
Baseline demographic characteristics of JUSTICE study participants (n = 345).

### Factor Analysis of the Civil Legal Needs Measure

The Kaiser-Meyer Olkin measure of sampling adequacy (KMO=0.68) and Bartlett’s test of sphericity (p<0.001) suggested that our sample was suitable for a factor analysis. Our exploratory factor analysis initially grouped our 18 items into seven latent factors, but two factors contained only one item and thus were excluded from subsequent analyses. The five remaining latent factors accounted for 50.5 percent of the total variance. After reviewing the items contained in the five latent factors, we added items to two latent factors based on our assessment of the broader literature on social determinants of health. Table 3 shows the final factorization of the 18 civil legal needs. The final categories were debt, public benefits, child support, housing, and healthcare access.^17^

**Table 3:**
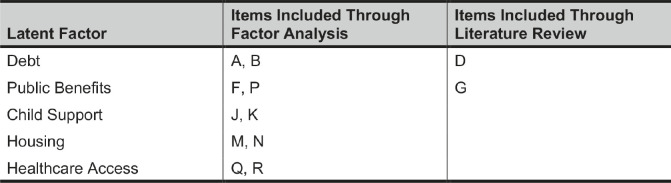
The five latent factors generated by factor analysis of the 18 civil legal needs and a literature review. Letters correspond to the civil legal needs listed in Table 1.

Table 4 shows the prevalence of each civil legal need category among each demographic group of participants. Participants who identified as White (55.1%), as some other race or ethnicity (57.1%), and as female (65.5%) were more likely than not to report debt-related needs. Female participants (55.2%) and those who had gone over 24 hours without food because they could not afford it (50.7%) were more likely than not to report needs related to public benefits. Overall, needs related to child support, housing, and healthcare access were less commonly reported, but their relative prevalence did vary with respect to certain demographic features. Hispanic participants (25.9%) were the most likely of any racial or ethnic group to report needs related to child support. And female participants (24.1%) were more likely than male participants (6.6%) to report housing-related needs, while participants living in transitional homes (4.9%) were less likely than participants in other housing arrangements to report housing-related needs.

**Table 4:**
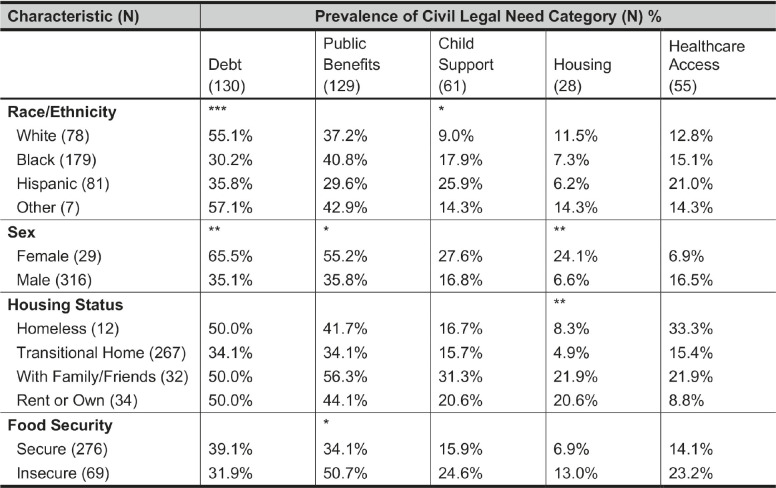
The percentage of JUSTICE study participants in a given demographic category who reported having at least one post-incarceration civil legal need in a given need category. Asterisks denote statistical significance based on Pearson’s chi-squared test. Numbers in parentheses indicate the total number of participants in that demographic category or with that post-incarceration civil legal need.

To provide sharper context for the associations we test in the following section, we translate the relative prevalences reported above into the demographic differences between participants with a given category of civil legal needs and participants without it. Participants with debt-related needs were more likely to be White (33.1% vs. 16.3%) and female (14.6% vs. 4.7%), and less likely to be Black (41.5% vs. 58.1%). Participants with needs related to public benefits were more likely to be female (12.4% vs. 6.0%) and food-insecure (27.1% vs. 15.7%). Participants with needs related to child support were more likely to be Hispanic (34.4% vs. 21.1%). And participants with housing needs were more likely to be female (25.0% vs. 6.9%) and less likely to be in a transitional home (46.4% vs. 80.1%).

### Chi-Squared Tests of the Associations Between Outcomes and Latent Factors

Table 5 reports the results of our chi-squared tests of the associations between the five latent factors of civil legal needs and the three outcomes. Moderate to severe psychosocial stress was 11-34 percentage points more prevalent among participants with civil legal needs related to debt, public benefits, housing, or healthcare access. The association between the housing factor and psychosocial stress is especially pronounced: 92.9 percent of JUSTICE study participants who reported at least one housing-related need also reported stress, compared to 58.9 percent of participants who did not report any housing-related needs. While several civil legal factors are positively associated with stress, we find no significant associations with uncontrolled high blood pressure or uncontrolled high cholesterol at baseline.

**Table 5:**
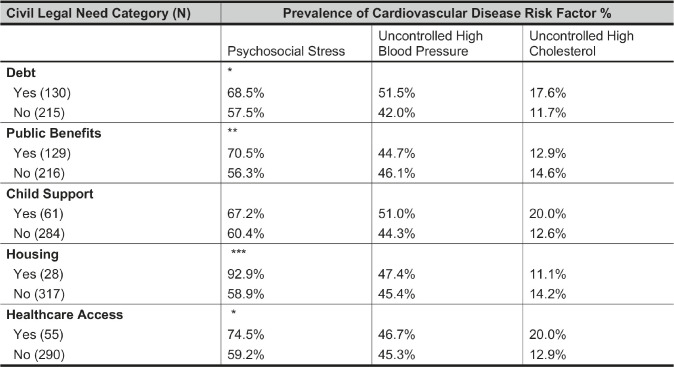
Baseline prevalence of psychosocial stress and uncontrolled CVD risk factors among JUSTICE study participants who reported each of five civil legal needs, compared to JUSTICE study participants who did not. Asterisks denote statistical significance based on Pearson’s chi-squared test. Associations are measured contemporaneously; future analysis of the complete JUSTICE study data might determine how these associations evolve over time.

## Discussion

Our analysis suggests a positive association between several civil legal need categories and psychosocial stress. We found that study participants with civil legal needs related to debt, public benefits, housing, or healthcare access were more likely to have moderate to severe psychosocial stress at baseline compared with those without civil legal needs. Research has shown that such stress, whether acute or chronic, is a significant predictor of CVD,^18^ of the same magnitude as traditional CVD risk factors.^19^

The null associations with uncontrolled high blood pressure and uncontrolled high cholesterol merit further research. It could be that civil legal needs do not influence CVD risk factor control, or there might be a lag between the development of civil legal needs and measurable changes in these CVD risk factors; blood pressure and cholesterol might worsen over time with the chronic stress of a persistent civil legal need. Since our data are cross-sectional, measured at baseline within three months of release from incarceration, they cannot distinguish between these two competing explanations. But future analysis of the complete, longitudinal JUSTICE study data might be able to do so, in addition to shedding light more generally on how the associations between civil legal needs and CVD risk factors trend over time.

Our work also identifies potentially high-impact ways for practitioners to promote patients’ cardiovascular health. For example, the association between housing and psychosocial stress is particularly strong in our data. This suggests that, among the many civil legal needs of patients recently released from incarceration, prioritizing those related to housing might best reduce patients’ psychosocial stress and any potential downstream health risks. We caution, however, that more research is needed to validate the strength of the association and, importantly, establish causation.

More broadly, our analysis adds to the growing literature on the need to screen for civil legal needs in primary care and the role of medical-legal partnerships (MLPs) in healthcare. MLPs are a model for healthcare delivery whereby medical and legal professionals work collaboratively to address a full spectrum of civil legal needs affecting individual and community health. MLPs have been shown to decrease rates of secondary and tertiary healthcare utilization, including emergency department visits and hospital admissions.^20^ They have also been shown to improve patients’ financial security by helping them procure public benefits^21^ and stable housing.^22^ Additionally, MLPs have led to improvements in self-reported patient satisfaction and compliance with medical care,^23^ as well as improvements in stress levels and mental health.^24^ Our findings suggest that MLPs dedicated to serving recently released people might be particularly effective at promoting mental and cardiovascular health by reducing the high prevalence of civil legal needs among this specific patient population. Future research could assess how effectively MLPs resolve the specific civil legal needs of recently released people and improve health outcomes.

## Conclusion

The collateral consequences of incarceration that affect people even after they have served their sentence, some of which are statutory, can lead to civil legal needs related to debt, public benefits, housing, or healthcare access. We find a positive contemporaneous association between such needs and psychosocial stress, but not uncontrolled high blood pressure or uncontrolled high cholesterol, shortly after release. Future analyses of longitudinal data can help clarify whether associations with uncontrolled high blood pressure and uncontrolled high cholesterol do not exist or simply take time to materialize. But because psychosocial stress itself is already a modifiable risk factor for CVD, interventions that resolve the civil legal needs of people returning to the community after incarceration might also promote their cardiovascular health if the associations we identified are not merely correlational but also causal. Our analysis also suggests that certain categories of needs, like housing, might have an especially close connection to mental and cardiovascular health.
